# Supplementary material for: High-resolution differentiation and identification of the clinically relevant pathogens Haemophilus influenzae and Haemophilus aegyptius: combined whole-cell MALDI-TOF MS and nano-LC-MS/MS proteotyping for defining protein biomarkers
Source: Microbiol Spectr. 2026 Mar 30;14(5):e03542-25. doi: 10.1128/spectrum.03542-25 (PMC13141906; doi:10.1128/spectrum.03542-25)
Supplement: Supplemental material — Optimization of analytical procedures; Fig. S1 and S2. [file spectrum.03542-25-s0001.docx]

**Optimization of analytical procedures**

The MALDI-TOF MS identification approach targets proteins, mainly ribosomal, structural, and housekeeping proteins, which have substantial expression, a typical molecular weight range (m/z: 2,000–20,000), and a high pI, and are unaffected by external factors. Our preliminary experiments revealed that information content prerequisites should be considered when distinguishing very closely related species, such as having a significant number of biomarker peaks and balancing them between the low-molecular-mass and high-molecular-mass (>10 kDa) regions. The information gained from larger molecules (ranging from 10,000 to 40,000 Da) is considerably increased. This range is especially important since larger proteins tend to mutate more often, increasing the likelihood of detecting unique biomarkers that help differentiate closely related bacteria. It is also crucial to ensure reproducibility, mass accuracy, proper sample preparation, and pre-analytical procedures. The simplest processes that could be automated to produce MS data with high information content were evaluated, including the type and concentration of the matrix, sample preparation methods, the solvent mixture for dissolving the matrix, the amount of acid added, and measurement variables like laser energy and the total number of shots per sample. Data were initially collected using a CHCA matrix solution in a mixture of 50% acetonitrile and 2.5% TFA over a wide m/z range, with the highest density of mass ions found between 2,000 and 10,000 Da. As a result, matrices with different properties, such as DHB and SA, were tested with various solutions (50:50 [v/v] acetonitrile: 0.1% TFA in water or 70:30 [v/v] acetonitrile: 0.1% TFA in water) at different concentrations and levels of acidity to enhance signal reproducibility and peak intensities, especially in the high-molecular-mass (>10 kDa) region. DHB produced better peak intensities and fewer matrix interferences at lower acid concentrations (0.1% TFA), while SA was more suitable at higher acid concentrations (0.6% TFA). The optimal concentrations determined were 10 mg/mL for DHB and 25 mg/mL for SA. The heterogeneous crystallization pattern of DHB, which only formed crystals near the edge of the target well, made it less suitable for automated measurements (Figure 1). Additionally, using DHB resulted in significantly fewer peaks, particularly in the higher molecular mass range. Once these conditions were established, the double-layer matrix solutions of sinapinic acid (SA) were carefully tested. Two types of SA solutions were used: a saturated solution in ethanol and a saturated solution in TA50 solvent (50:50 [v/v] acetonitrile: 0.1% TFA in water). Due to enhanced ionization and analyte desorption, SA yielded protein signals with higher intensities and better resolution.

The best cell disruption methods to enhance the detection of *Haemophilus* spp. MALDI-TOF MS specific profiles for high molecular mass peaks were evaluated. The first method (whole-cell MALDI-TOF MS) involves applying individual colonies directly onto the MALDI target as a thin film in saturated SA solution in absolute ethanol, which is then immediately mixed with SA TA50. The second method includes extracting bacterial proteins using a solution of H2O:ACN:FA (15:50:35 or 80:10:10), followed by drying the cell-free extract and adding an optimized matrix solution. When using the H2O:ACN:FA (80:10:10) extraction protocol, the resulting spectra were clear and reproducible with higher resolution and significant mass signal intensities. However, suspensions of cells in H2O:ACN:TFA (15:50:35) prior to extraction were not effective for detecting peaks in the higher mass range, yielding only a partial set of peaks. These comparison results indicate that smearing cells directly onto the target, as described here, did not compromise the automatic acquisition of mass spectra (Figure 2). Furthermore, higher signal-to-noise ratios for specific protein signals suggested the presence of spectral "sweet spots,' likely due to optimal conditions at those spots, such as ideal analyte and matrix molecule distribution, which enhances ionization efficiency (Šedo et al. 2011).

Reference:

Šedo, O., Voráč, A. & Zdráhal, Z. Optimization of mass spectral features in MALDI-TOF MS profiling of *Acinetobacter* species. Syst Appl Microbiol 34, 30–34 (2011).

| 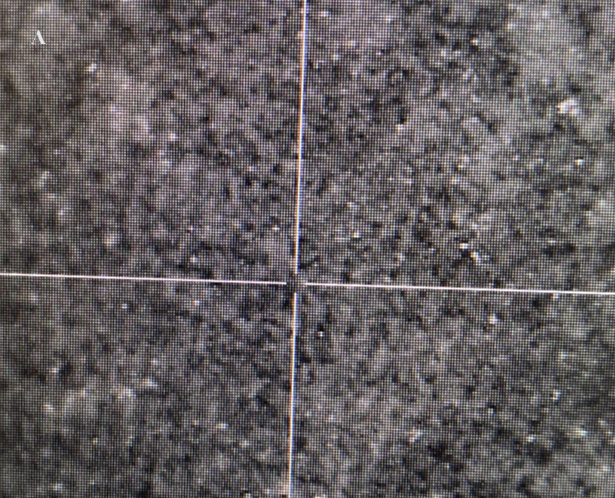 | 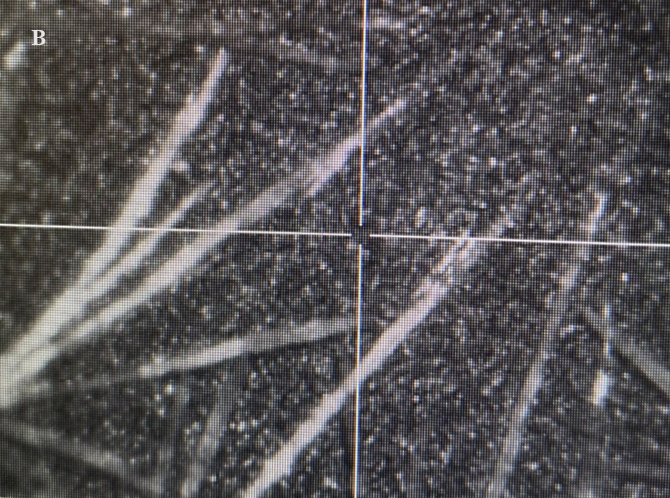 |
| --- | --- |

**Figure 1. (A)** Representative SA double layer spot, resulting in homogeneous crystallization due to the matrices’ ability to facilitate the incorporation of analytes into its crystalline structure. **(B)** The 2,5-DHB dried droplet spot suffers from heterogeneous crystallization, which primarily occurs at the edges of the target well, limiting its suitability for automated measurements and leading to fewer detectable peaks, particularly for high-molecular-mass analytes (>10 kDa).

| **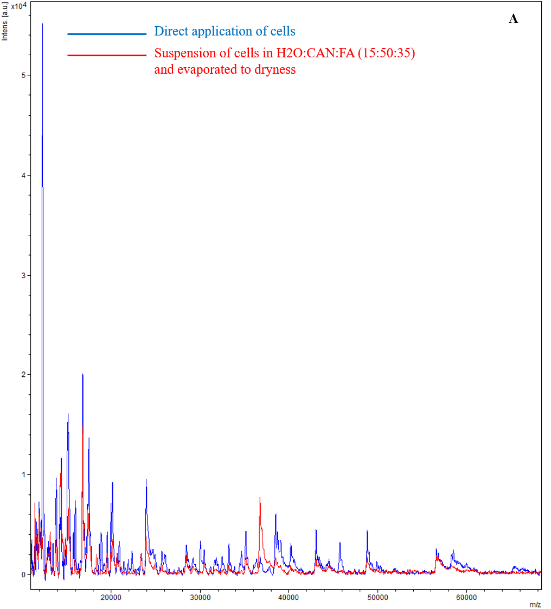** | **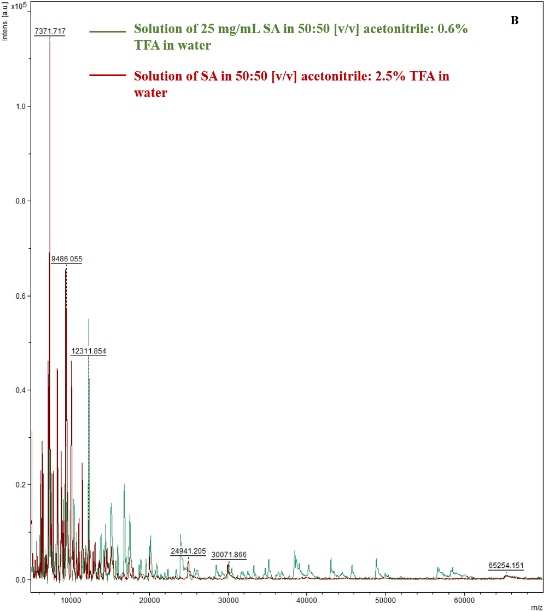** |
| --- | --- |

**Figure 2.** MALDI-TOF MS profiles for molecular-mass 3,000- 40,000 Da, showing the influence of the sample treatment method (A), and various levels of acidity (B), on *H. influenzae* CCUG 23945^T^.
